# Aquatic Exercise and Land Exercise Treatments after Total Knee Replacement Arthroplasty in Elderly Women: A Comparative Study

**DOI:** 10.3390/medicina57060589

**Published:** 2021-06-08

**Authors:** Chang-Hyung Lee, In-Hye Kim

**Affiliations:** 1 Department of Rehabilitation Medicine, Pusan National University Yangsan Hospital, Pusan National University School of Medicine, Yangsan 50612, Korea; aarondoctor@gmail.com; 2 Department of Rehabilitation Medicine, Pusan National University Yangsan Hospital, Yangsan 50612, Korea

**Keywords:** total knee replacement arthroplasty, hydrotherapy, rehabilitation

## Abstract

*Background and Objectives*: Early intensive exercise after total knee replacement arthroplasty (TKRA) has become increasingly popular due to its ability to enhance knee physical function and reduce pain. When implemented exclusively, aquatic exercise (AE) appears to be more advantageous than land exercise (LE), particularly in the early phase after TKRA. Our study aimed to compare the clinical efficacy of AE and LE with respect to their effects on pain and physical function after TKRA. *Materials and Methods*: Between February 2008 and January 2020, 100 female patients who underwent TKRA were enrolled in this retrospective study. We measured the range of motion (ROM) of the knee, the isokinetic strength of the knee joint (function), and pain both initially and one month after TKRA. Two weeks after TKRA, the participants were enrolled in either the AE or the LE program for a total of two weeks. Two 30 min sessions of intensive ROM and knee strengthening exercises and balance training were provided to the AE and LE groups for 10 days. The home exercise group (HE) only received information on ROM and strengthening exercises. There were 33, 21, and 46 patients allocated to the AE, LE, and HE groups, respectively. *Results*: The ROM of the side on which surgery was performed improved significantly in all groups, as did the pain scores. In the AE group, the knee flexor strength showed a tendency toward improvement. Contrastingly, there was no significant improvement in the knee extensor strength in the AE group. *Conclusions*: Overall, the AE and LE groups showed superior outcomes compared with HE. In addition, the AE group demonstrated some improvement in knee muscle strength even with a short hospital stay. Further study with long-term follow-up should be performed to better define the outcomes.

## 1. Introduction

Total knee replacement arthroplasty (TKRA) is a reliable treatment for degenerative knee arthritis. It is also the most commonly performed musculoskeletal surgery [1,2,3]. Although several studies have shown that pain and quality of life improve after TKRA, a standard postoperative rehabilitation treatment method is yet to be established [4]. As TKRA has become more common, the need for rehabilitation treatment that allows for a quick return to daily life after the surgery has also been highlighted. Initiation of a rehabilitation treatment immediately after surgery has become increasingly common, and treatment may be performed as inpatient and outpatient care according to the patient’s preference. Physiotherapy after TKRA focuses on exercising to increase muscle strength and endurance through the use of treatment modalities and gait training that improve overall quality of life and physical function [5,6,7,8,9]. 

As rehabilitation treatment after TKRA has become more common, several methods of rehabilitation treatment have emerged, one of which is aquatic exercise (AE). Postoperatively, patients are not able to perform full-weight bearing due to pain and edema of the knee joint. According to a previous study, standard rehabilitation care after TKRA includes the use of modalities such as ice, heat, and massage, and the exercise protocol involves range of motion (ROM) and strengthening exercises [9]. AEs are widely used in rehabilitation to improve mobilization and muscle strengthening by harnessing the characteristics of water [10,11]. The buoyant force of water reduces the load on the musculoskeletal system. The drag force of water can be modulated by movement velocity and surface area [10]. In addition, the viscous resistance of water can increase joint stability and improve the strength of the surrounding muscles. Thus, due to the nature of water, hydrotherapy can reduce swelling and relieve pain in TKRA patients. Its added benefit is that it allows for the safe reduction of body weight during exercise [12,13,14,15,16,17,18].

These beneficial aqua therapies have been used to perform rehabilitation and yield favorable outcomes [19,20]. However, there is a lack of quantitative comparative studies that discuss the appropriate exercise intensity for AE and land exercise (LE) [6,21]. Most studies have only evaluated the postoperative outcomes, and patients’ symptoms were compared using subjective pain and functional scores; thus, objective physical improvements could not be identified. In these studies, during the postoperative rehabilitation period, patients were educated about knee muscle ROM and strengthening exercises, pain reduction, and improvement of functional outcomes. To objectively evaluate the efficacy of AE and LE, pre-and post-treatment evaluations using objective measurements of physical function should be undertaken. Thus, the aim of this study was to quantify and evaluate the differences between AE and LE treatments after TKRA.

## 2. Materials and Methods

### 2.1. Participants

A total of 100 patients (only women; mean age, 73.17 ± 7.151 years) who underwent TKRA between 2008 and 2020 were included in this study. According to a computer-based allocation program, the patients were randomly divided into the following three groups: AE, LE, and home exercise (HE). However, if the patients did not agree to AE due to individual preferences or if they could not perform either AE or LE, they were placed into one of the other groups. 

The study procedures were reviewed and approved by our hospital’s institutional review board (No. 05-2020-233). We confirm that all applicable institutional and governmental regulations concerning the ethical use of human volunteers were followed during the course of this study. The need for informed consent was waived due to the retrospective nature of the study.

### 2.2. Assessment Method

#### 2.2.1. Passive Knee Joint ROM and Pain Measurement

A well-trained physiatrist measured patients’ knee joint ROM bilaterally. Passive knee ROM was measured using a long-arm universal goniometer in the supine position [22,23]. Pain was measured using a visual analog scale (VAS), which ranged from 0 to 10 (0, no pain; 10, excruciating pain). Using this VAS scale, we recorded the patients’ subjective scores the day before the surgery and 4 weeks postoperatively [24].

#### 2.2.2. Isokinetic Measurement of the Knee Joint Strength 

The maximum concentric strength of the knee muscles was measured using an isokinetic exercise device (Biodex Medical Systems, Inc., Shirley, NY, USA). To minimize errors, the instrument was calibrated before and after each measurement. The patient was educated on the measurement method, and the hip joint and thigh were fixed and measured. The lever arm of the isokinetic device was fixed at 2.5 cm above the lateral malleolus, and the axis of knee joint rotation was aligned with the lateral condyle of the femur. 

The participants were seated in the dynamometer, adopting a standardized position of 85° hip flexion from the anatomical position. The hip and thigh were stabilized with belts. During the test, the participants were instructed to keep their arms crossed with each hand resting on the opposite shoulder. The full range of motion of the knee was measured. The healthy leg was measured first; however, in case of bilateral knee surgery, the examination was first performed on the right side. After 2–3 sets of submaximal flexion and extension movements, a maximum of 5 flexion and extension movements were performed at an angular velocity of 60°/s, with 2 min intervals between trials. Data were collected using a sampling rate of 100 Hz, and these were subsequently analyzed with the software Acqknowledge, version 4.1 (Biopac Systems, Inc., Goleta, CA, USA). Each individual curve was inspected to identify true isokinetic torques within a 95% confidence interval of the angular velocity of 60°/s [25,26].

The examiner was encouraged to obtain the peak torque value by providing instructions to the patient while the measurements were being taken; thus, the peak torque of the knee flexion and extension data were measured.

#### 2.2.3. Exercise Protocols for AE, LE, and HE 

All the patients in the AE and LE groups started treatment 10 days after TKRA. The same physiotherapist conducted the treatment for the AE and LE groups. The treatment duration for each session was 30 min, and the sessions occurred 5 times per week for 2 weeks (a total of 10 sessions). 

The AE treatment was conducted in warm, deep water (mean temperature, 31.5 °C). This allowed the water level to be at approximately the xiphoidal level for each participant (1.2–1.5 m) in a 25 m pool. Immediately before immersion, a waterproof dressing was applied to the surgical site on each patient’s knee. Each session included repetitions of walking forward and backward, stepping sideways, jogging, jumping, kicking, and performing step-ups, knee ROM exercises, lunges, combined squats, and upper extremity exercises [27]. The exercise intensity was set at a modified Borg scale value of 7 [28] and was increased until the last session according to how the patients adapted. The LE treatment consisted of 10 min of cycling on a stationary ergometer, 10 min of walking on a motor-driven treadmill, isometric standing, balance, and knee ROM exercises at a bar, and 10 min of sit-to-stand exercises from chairs of varying heights [29]. As for the AE group, the exercise intensity was set at a modified Borg scale value of 7 and increased according to patients’ tolerance.

In addition, all the patients in the AE and LE groups received general pain interventions that included superficial heat and gentle passive ROM exercises for 30 min during the inpatient period. If there were any instances of complication or pain aggravation during AE or LE, the patient was recommended to stop the exercise.

For the HE group, the patients were educated on ROM exercises, knee joint strengthening exercises, and light walking before they were discharged. Initially, the exercises were of minimal intensity (scale 2–3) according to the modified Borg category scale, which was translated into Korean, and their intensity was gradually increased to achieve high levels (scale 6–7). Outpatient adherence to exercise was assessed, along with popular comfortable and highest intensity as per the subjective intensity level [21]. 

### 2.3. Statistical Analysis

The collected data were analyzed using a two-tailed test with a significance level of 0.05. SPSS version 20.0 for Windows (SPSS Inc., Chicago, IL, USA) was used for the analysis. Between-group differences in the baseline demographic, anthropometric, and surgical data were analyzed using one-way analysis of variance or the Chi-squared test for independent dichotomous data. In the presence of statistically significant differences, post hoc analysis was performed using the Bonferroni’s method. The outcomes, such as ROM and pain scores, were analyzed using analysis of covariation (ANCOVA). A *p*-value of < 0.05 was considered to be statistically significant. To compare the pre- and postoperative knee joint muscle strength, a paired *t*-test was used for the AE group due to its normal distribution, while a Wilcoxon signed-rank test was used for the LE group because it did not follow a normal distribution. The Kolmogorov–Smirnov normality test was performed to check the normal distribution of the variables.

## 3. Results

The patients’ demographic and descriptive data are shown in Table 1. A total of 100 women participated in this study. There were 33, 21, and 46 patients that were allocated to the AE, LE, and HE groups, respectively. With respect to the patients’ demographic characteristics, there were no statistically significant differences between any of the groups, except in relation to age. Patients in the HE group were significantly younger than those in the AE and LE groups (*p <* 0.001). The mean ages were 75.24 ± 5.995 years, 77.67 ± 8.534 years, and 69.63 ± 5.401 years in the AE, LE, and HE groups, respectively. As a significant difference in age was observed, post hoc analysis was performed using the Bonferroni’s method, which confirmed the difference.

Table 2 shows the differences in the pre- and postoperative knee joint ROM and pain scores in each exercise group. The pain scores improved in all groups; however, this was not statistically significant according to ANCOVA. (F = 2.606, *p* = 0.110). The ROM of the operated knee improved in all groups, and the changes in these results were statistically significant (F = 5.424, *p* = 0.021). In addition, improvement of the ROM of the normal side was observed in all exercise methods, with statistically significant between-groups differences.

Table 3 shows the changes in knee joint muscle strength in the AE and LE groups. In the AE group, the knee flexor strength increased slightly from 20.867 ± 11.917 to 21.154 ± 11.600; however, this difference was not statistically significant (*p* = 0.874). Moreover, there was no change in the knee extensor strength after treatment (*p* = 0.660). We could not compare the size difference before and after treatment in the LE group using nonparametric tests. Because the sample size was small, only the difference in muscle strength before and after the treatment could be confirmed, and this was also not statistically significant. Since body weight may contribute to differences in muscle strength, the measured value was divided by the body weight and analyzed; however, the difference remained not statistically significant. 

No significant side effects were reported during the hospital stay in the AE and LE groups. Only the HE group reported three adverse effects that included wound dehiscence and surgical site infection. 

## 4. Discussion

Although intensive rehabilitation treatment could be ideal for TKRA patients, the implementation of early intensive exercise may be difficult due to pain and swelling immediately after surgery. Aqua therapy has been used in various therapeutic fields to help manage conditions such as arthritis, neuromuscular diseases, and cerebral palsy [10,16,30,31,32,33]. The main advantages of aqua exercise for the patients are the lifting force (buoyancy) and density, hydrostatic pressure, viscosity, and thermodynamics of water [34]. The beneficial effects of aqua treatment have allowed patients with impaired motor function to perform exercises both efficiently and independently [35]. 

In addition, water has greater thermal conductivity than the equivalent volume of air [36]. Water temperatures can substantially suppress or stimulate body processes and reactions to various stimuli. Water can also assist in pain relief and swelling reduction. Viscosity refers to the resistance to the flow of a fluid during motion. A limb that is moved relative to water is subject to its resistive effects, which are called drag force and turbulence. Buoyancy also decreases the weight placed on a joint, reduces pain, and enables movements that could not otherwise be performed during land-based exercises. Water turbulence can increase exercise load by creating resistance, and this can be adjusted to control exercise intensity [37]. Hydrostatic pressure reduces swelling and facilitates movement. Therefore, due to the nature of water, AE is an ideal therapy after TKRA.

All study groups, including the AE group, demonstrated improvements in pain and knee joint ROM after TKRA. In our study, the participants in the AE and LE groups were older than those in the HE group (mean ages were 74.11 ± 6.94 years and 77.64 ± 8.33 years in the AE and LE groups, compared to 69.61 ± 5.50 years in the HE group), and treatment for the HE group only started 10 days after TKRA. Additionally, the hospital rehabilitation period was limited to two weeks. This may not have been long enough to induce a significant change with respect to AE or LE. Despite its limited conditions, there were changes in pain and ROM after applying the intensive rehabilitation program. In this way, although there were no significant changes noted (including in the knee strength), we observed that aqua therapy that effectively harnessed the characteristics of water could be used safely and efficiently in the early period after TKRA. 

In general, hamstring strength can be improved through exercises of moderate to high intensity, such as stair climbing [38]. In our report, the patients’ initial lower extremity strength (knee extensor and flexor strength) was very low due to previous pain and deconditioning. As patients could not walk or exercise, lower extremity weakness (especially in the hamstring muscle) was observed. Thus, efficient hamstring strengthening could not be attained through LE or daily activities under these physical conditions. Contrastingly, due to the characteristics of water (such as viscosity and turbulence), under aquatic conditions, comprehensive strength improvement (including knee extensor/flexor strength) could be achieved, even when walking.

There are several possible explanations that may account for the improvements in pain. Improvements in hip and knee muscle strength partially account for the improvement in knee pain through support of the loaded force on the knee joints. The characteristics of water, such as its thermodynamics and hydrostatic pressure, affect pain relief [39]. Hydrostatic forces reduce lower extremity swelling. In water, changes in the autonomic and circulatory systems increase the blood flow and improve muscle and tissue healing [40]. Moreover, through buoyancy, joint loading and pain are reduced [41]. This study demonstrated that AE and LE had beneficial effects on patients in the early period after TKRA. When considered exclusively, AE may be an effective and safe treatment modality for the early period after TKRA. 

The suitability of AE in the early phase after TKRA may be due to the reduction in joint loading and subsequent pain relief. Compared to the other interventions, AE was shown to be safe and effective, and our study established that it was not inferior to other exercise methods.

However, there are several limitations to our study. First, participants were not randomly assigned to each group in a precise manner. Some patients preferred to not undertake AE, and more of the younger patients were allocated to the HE groups. In addition, this study was only on women. There may be differences in results according to patients’ sex. Second, we evaluated the patients’ pre-/post-functional statuses in a short treatment period. This may not have been long enough to evaluate the long-lasting effects of AE or LE. A longer treatment duration with long-term follow-up should be undertaken. Moreover, other confounding factors such as lifestyle, working conditions, and medication use should also be considered after treatment. Third, other factors should be measured in addition to ROM, pain, and muscle strength. Swelling, lymphatic flow, motivation to change, pain threshold, functional scale, and other hormonal changes should also be evaluated to determine whether AE is a superior rehabilitation method. Fourth, the patients’ initial knee joint muscle strength was lower than average [42,43]. This may have been due to the patients’ longstanding deconditioned state. 

## 5. Conclusions

AE and LE could be appropriate rehabilitation methods in the early period after TKRA. In addition, AE appears to be an ideal treatment method to increase muscle strength even with a limited hospital stay. Further studies should be performed with longer treatment sessions, long-term follow-up, and consideration of other functional factors to determine the generalizability of our findings.

## Figures and Tables

**Table 1:** Comparison of the groups’ characteristics.

Variables		AE	LE	HE	*p*-Value
		(*n* = 33)	(*n* = 21)	(*n* = 46)	
Age (years)		75.24 ^a^ ± 5.995	77.67 ^a^ ± 8.534	69.63 ^b^ ± 5.401	<0.001 **
Body weight (kg)		63.469 ± 9.752	61.053 ± 9.897	63.555 ± 9.315	0.579
Height (cm)		150.157 ± 7.016	152.504 ± 6.014	153.158 ± 5.756	0.107
BMI (kg/m^2^)		28.103 ± 3.519	26.255 ± 4.079	27.117 ± 3.889	0.214
Surgical side	Left	3 (9.09%)	5 (23.8%)	10 (21.7%)	0.144
	Right	2 (6.06%)	3 (14.2%)	4 (8.69%)	
	Both	28 (84.8%)	13 (61.0%)	32 (69.56%)	

AE, aquatic exercise; LE, land exercise; HE, home exercise; BMI, body mass index. ** *p*-value < 0.001. Values are expressed as the mean ± standard deviations or number (%). ^a,b^ Means followed by the same letters are not statistically different as determined by the post hoc Bonferroni’s method at *p* = 0.05.

**Table 2:** Differences in knee joint ranges of motion and pain scores before and after surgery in each exercise group.

Variable		AE		LE		HE		F	*p*-Value
		Before	After	Before	After	Before	After		
ROM(°)	Surgical side	118.209 ± 17.724	125.955 ± 11.785	119.166 ± 20.301	126.944 ± 12.087	116.024 ± 17.050	123.253 ± 13.602	5.424	0.021 *
	Normal Side	126.000 ± 12.942	126.000 ± 12.942	133.333 ± 14.790	134.444 ± 9.824	134.000 ± 10.555	134.333 ± 12.658	17.356	<0.001 ***
Pain score		5.000 ± 1.531	2.390 ± 1.420	4.950 ± 1.527	2.360 ± 1.217	4.570 ± 1.354	3.900 ± 1.373	2.606	0.11

ROM, range of motion; AE, aquatic exercise; LE, land exercise; HE, home exercise. * *p*-value < 0.05 and *** *p*-value < 0.001. Values are expressed as the mean ± standard deviations. Covariate: ROM; pre-surgical knee joint range of motion, pain score; pre-surgical pain score.

**Table 3:** Comparison of the knee joint muscle strengths in the AE and LE groups. (All the patients who performed this test underwent bilateral TKRA).

Variable		Before	After	*p*-Value
AE (*n* = 24)	Extension (Nm)	32.396 ± 21.073	30.979 ± 20.618	0.660
	Flexion (Nm)	20.867 ± 11.917	21.154 ± 11.600	0.874
LE (*n* = 8)	Extension (Nm)	31.500 (20.925–62.850)	29.150 (23.050–40.175)	0.401
	Flexion (Nm)	19.100 (11.315–31.825)	23.750 (8.425–31.225)	0.889
AE (*n* = 24)	Extension (Nm/kg)	0.489 ± 0.332	0.462 ± 0.285	0.632
	Flexion (Nm/kg)	0.319 ± 0.179	0.322 ± 0.166	0.931
LE (*n* = 8)	Extension (Nm/kg)	0.498 (0.309–1.079)	0.498 (0.412–0.636)	0.674
	Flexion (Nm/kg)	0.304 (0.175–0.528)	0.413 (0.149–0.511)	0.889

AE, aquatic exercise; LE, land exercise. Values are expressed as the mean ± standard deviation or median (Q1–Q3). As the AE group showed normal distribution by the Kolmogorov–Smirnov test with weight, a paired t-test was performed. As the LE group showed non-normal distribution weight, the Wilcoxon signed-rank test was performed.

## Data Availability

All the data are available from the corresponding author upon reasonable request.
